# Chest imaging representing a COVID-19 positive rural U.S. population

**DOI:** 10.1038/s41597-020-00741-6

**Published:** 2020-11-24

**Authors:** Shivang Desai, Ahmad Baghal, Thidathip Wongsurawat, Piroon Jenjaroenpun, Thomas Powell, Shaymaa Al-Shukri, Kim Gates, Phillip Farmer, Michael Rutherford, Geri Blake, Tracy Nolan, Kevin Sexton, William Bennett, Kirk Smith, Shorabuddin Syed, Fred Prior

**Affiliations:** 1 Department of Radiology, University of Arkansas for Medical Sciences, Little Rock, Arkansas USA; 2 Department of Radiology, University of Texas, Southwestern Medical Center, Dallas, Texas USA; 3 Department of Biomedical Informatics, University of Arkansas for Medical Sciences, Little Rock, Arkansas USA; 4 Department of Surgery, University of Arkansas for Medical Sciences, Little Rock, Arkansas USA

**Keywords:** Sequence annotation, Translational research

## Abstract

As the COVID-19 pandemic unfolds, radiology imaging is playing an increasingly vital role in determining therapeutic options, patient management, and research directions. Publicly available data are essential to drive new research into disease etiology, early detection, and response to therapy. In response to the COVID-19 crisis, the National Cancer Institute (NCI) has extended the Cancer Imaging Archive (TCIA) to include COVID-19 related images. Rural populations are one population at risk for underrepresentation in such public repositories. We have published in TCIA a collection of radiographic and CT imaging studies for patients who tested positive for COVID-19 in the state of Arkansas. A set of clinical data describes each patient including demographics, comorbidities, selected lab data and key radiology findings. These data are cross-linked to SARS-COV-2 cDNA sequence data extracted from clinical isolates from the same population, uploaded to the GenBank repository. We believe this collection will help to address population imbalance in COVID-19 data by providing samples from this normally underrepresented population.

## Abstract (summary)

**Table:** 

Measurement(s)	COVID-19 image features • chest • viral genome
Technology Type(s)	Radiograph • computed tomography • whole genome sequencing
Factor Type(s)	COVID-19 diagnosis
Sample Characteristic - Organism	Homo sapiens • SARS-CoV-2
Sample Characteristic - Environment	rural area
Sample Characteristic - Location	United States of America

Machine-accessible metadata file describing the reported data: 10.6084/m9.figshare.12980795

## Background & Summary

Rural Americans are at greater health risk and are more susceptible to five leading causes of death than urban Americans^1^. Bolin *et al*.^2^ summarize findings of the Rural Healthy People 2020 survey and identify an array of health issues that increase the health risks faced by the rural population of the United States. Significant among these are higher rates of hypertension, obesity, heart disease, stroke, and substance abuse, coupled with limited access to quality healthcare and public health infrastructure. Arkansas (AR) is a rural state, with 42% of Arkansans living in rural counties, (https://worldpopulationreview.com/states/arkansas-population/) compared to 15% in the country as a whole. Arkansas’ health problems mirror those of the rest of the rural U.S^3^.

**Fig. 1:**
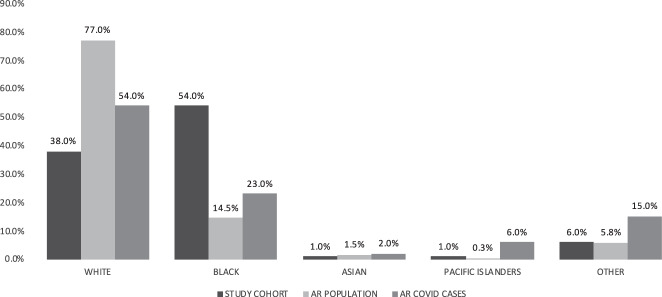
Study cohort population distribution. Racial characteristics of study cohort in comparison to Arkansas total population and the state’s Covid-19 cases.

According to recent American Census Survey (ACS) data, the composition of Arkansas’ population is white 77.0%; black, 14.5%; Asian, 1.5%; pacific islanders, 0.3%; and other 5.8% (other race, 2.6%; two or more races, 2.5%; native American, 0.7%), (Fig. 1). The study cohort reported here includes 105 COVID-19 patients seen at the University of Arkansas for Medical Sciences (UAMS) with the following demographics characteristics: 50% (n = 53) female and 50% (n = 52) male; age range 19 to 91 years; and white 38% (n = 40), black 54% (n = 57), Asian 1% (n = 1), pacific islanders 1% (n = 1), other 6% (n = 6), (Fig. 1). The study population is representative of neighboring, rural counties in the proximity of UAMS. Figure 1 also illustrates the demographics of the COVID-19 infected population in Arkansas as of July 20, 2020 based on statistics reported daily by the Arkansas Department of Health on their web site https://experience.arcgis.com/experience/c2ef4a4fcbe5458fbf2e48a21e4fece9.

Radiologic imaging has proven to be an essential tool for screening, diagnosis, and management of patients with COVID-19 infection^4,5^. In March 2020 the American College of Radiology published guidelines that recommended the use of chest radiography as the “first-line test to diagnose COVID-19,” with CT being used “sparingly and reserved for hospitalized, symptomatic patients”^6^. Largely because of these guidelines, the bulk of imaging studies performed thus far in the US are chest radiographs. The image collection presented here is consistent with these guidelines.

The ability to sequence the whole genome of the SARS-CoV-2 virus is essential for tracking the evolution of the virus and the resulting disease. GenBank^7^ contains 22,542 SRA runs and 11,142 Nucleotide records (as of July 20, 2020) and is growing daily. We have contributed to this repository sequences of the virus strains infecting our local population.

## Methods

### Patient selection

Images and clinical correlate data were obtained with approval of the UAMS Institutional Review Board (IRB number 228350) which includes open access publication of this data in de-identified form. All data were collected from hospitalized patients with a positive COVID-19 lab test (PCR) verified diagnosis who had imaging studies within eight days prior to diagnosis and at least one imaging study post diagnosis. Emphasis was placed on patients with multiple post diagnosis imaging studies. All imaging studies of the head were excluded due to issues of potential patient identification from volumetric CT reconstructions^8,9^.

### Imaging

**Fig. 2:**
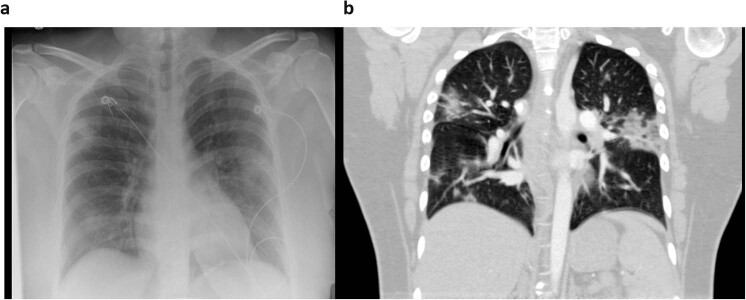
Chest Radiograph (**a**) and Computed Tomography (CT) image in the sagittal plane (**b**) of the same patient (Covid-19-Ar-16434363) taken on the same day. Patchy airspace opacities and ground-glass changes are seen in both lungs.

Imaging techniques were standard of care, primarily portable (97%), digital chest radiographs collected either by computed radiography, CR, (19 patients, 26 image series) or direct digital capture, DX, (100 patients, 236 series). CT (computed tomography) studies were performed on 21% (23) of patients depending on the severity of symptoms and clinical status (resulting in 199 CT image series). Radiographs were performed using a single view AP (anteroposterior) technique or PA (posteroanterior) and lateral views depending on patient mobility and intubation status. CT was performed on multi-slice, spiral scanners. Contiguous 1 mm axial images were obtained through the chest (Online-only Table 1 provides acquisition parameters for each of the 199 CT series). Axial (3 mm spatial resolution) and 2 mm sagittal and coronal reconstructions were performed and subsequently reviewed on a dedicated Picture Archive and Communication System (PACS) workstation. A total of 256 chest imaging studies were identified, including 233 radiographs and 23 CT studies performed on a total of 105 patients. Figure 2 is an illustrative example of a chest radiograph and CT for the same patient taken on the same day. Final radiology reports for these examinations signed by board-certified radiologists were analyzed for key imaging findings.

### Summary of key imaging findings

The most frequent pattern of imaging findings is ‘Organizing pneumonia’ which is essentially a pattern of lung changes as a response to inflammation. The reported key imaging findings on radiographs and CT include confluent and patchy multifocal airspace opacification, either ground-glass opacities or consolidation. These airspace opacities were predominantly bilateral, multifocal, and peripheral. Although more commonly, these changes were diffuse and bilateral, lower lobe predominance was seen in cases that were more focal. A total of 59/232 (25%) radiographs and 8/23 (35%) CT were negative for airspace opacification. In our patient population, when present, ground glass changes were more common than consolidation, likely suggesting an earlier presentation in the disease course. Pleural effusions were rare and, when present, were only trace to mild. Other atypical findings like mediastinal lymphadenopathy, cavitation, and pneumothorax were not identified in our patient population. One of the 23 CT studies showed bilateral segmental pulmonary emboli.

The majority of ICU patients (28/29) showed radiographic AP changes, with 59% showing bilateral diffuse changes with left lower lobe involvement. One Radiographic study for one ICU patient had no significant findings. Seventy percent (7/10) of ICU mortality occurred in patients showing left lower lobe opacities. In contrast, one of these patients had no significant findings on CR or DX radiographic studies, 1 had general atelectasis, and 1 had diffuse disease without left lower lobe opacity.

### Clinical correlates

The average age in the cohort was 54.3 years old (range 19–91) with an even sex distribution (52 Male, 53 Female). The worldwide incidence is reported to be close to 1:1, with a 50% increase in hospitalizations, ICU stays, and mortality among males^10^. The racial characteristics of the cohort are presented in Fig. 1 in comparison to the total population of Arkansas and the current characteristics of the state-wide infected population. The average BMI in the cohort is 33.1 (18.7–64.9), well within obese range (30.0 or higher). Key Comorbidities include burns (2%), malnutrition (3%), pregnancy (4%), chronic kidney disease (11%), diabetes (21%), organ transplant (3%), and cancer (24%).

**Fig. 3:**
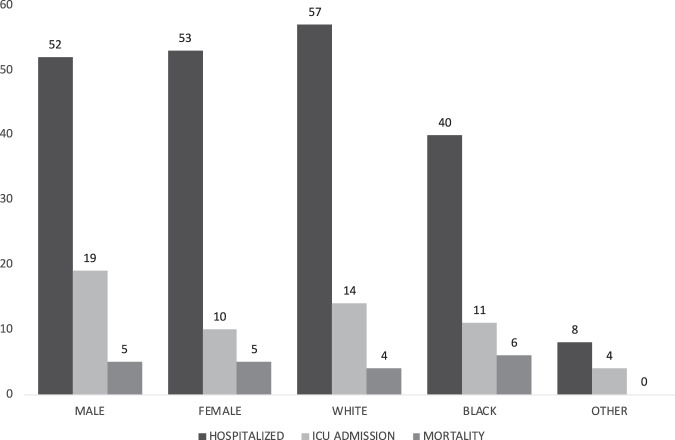
Disease Progression (by Sex, Race). Breakdown of the rates of hospitalization, ICU admission, and mortality by sex and race.

The overall ICU admission rate was 28% (29/105). The Average age among those admitted to the ICU was 58 (range 25–89), slightly higher than the average for the cohort as a whole. The racial breakdown of ICU admissions included 28% of the white patients, 25% of the black, 50% of other, and 100% of Pacific islanders. The ICU population was 66% male and 33% female and included 1 pregnant patient. Forty three percent of patients admitted to the ICU had BMI greater than 30. Of the black patients, 21% had diabetes and 29% chronic renal disease, while among white patients the highest percentage comorbidities were cancer (10%) and diabetes (10%). The ICU mortality rate was 34.4% (10/29) which is 1.5 times the national average of 23.6%^11^. The overall mortality rate was 10% (10/105) and all 10 patients in the mortality group were first admitted to ICU. Arkansan males were 1.95 times more likely to go to ICU (19/52 vs. 10/53). Our data shows an almost even overall mortality rate among males (5/53) and females (5/52). The data also suggest that once in the ICU, female mortality occurs at a rate 1.9 times that of males (5/19 vs. 5/10). Figure 3 summarizes rates of hospitalization, ICU admission and mortality stratified by sex and race.

### COVID-19 testing samples

A set of residual, de-identified nasopharyngeal samples testing positive for SARS-CoV-2 by quantitative reverse-transcriptase PCR, was obtained from the clinical molecular diagnostics lab at the University of Arkansas for Medical Sciences (UAMS). All samples were obtained with approval of the UAMS Institutional Review Board (IRB number 260840). This approval includes the right to publish viral sequences with all references to the specific human participants who provided the samples being removed.

### SARS-CoV-2 genome sequencing

**Fig. 4:**
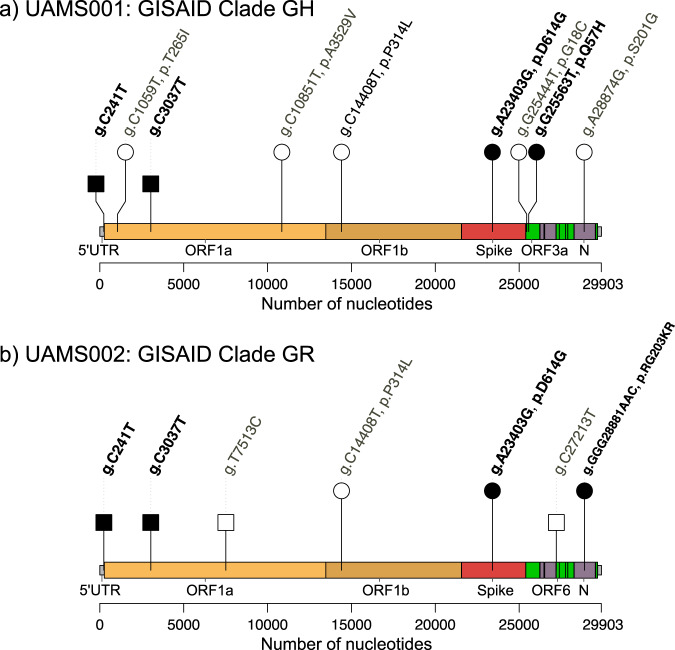
The illustration of SARS-CoV-2 genome organization and a plot of mutations. Two isolates of UAMS SARS-CoV-2 genome, UAMS001 (**a**) and UAMS002 (**b**), are grouped into clade G (D614G mutation) but different sub-group (GH and GR, respectively). Genome size of both isolates is 29,903-nucleotides. The leader sequence is represented by a small grey square at the 5′ terminus of the genome. Open reading frames (ORFs) 1a (yellow) and 1b (brown) encode the nonstructural proteins. Spike (red), envelope, membrane, and nucleocapsid are shown in purple and the accessory proteins in green. Vertical lines represent the presence of a mutation. Above each line, the variants are annotated as the nucleic acid change (such as g.C241T) and amino-acid change (such as p.D614G) at that specific site. The non-synonymous mutations are presented by circles and the synonymous mutations squares. Black circles and black squares are the locations used for clade identification characterized by Global Initiative on Sharing All Influenza Data (GISAID).

RNA extracted from nasopharyngeal samples was reverse transcribed, as described in protocol published by ARTIC Network (10.17504/protocols.io.bdp7i5rn). SARS-CoV-2 specific primer set version 3 (218 primers) was kindly provided by Dr. Joshua Quick, University of Birmingham. The PCR amplification and extension condition of SARS-CoV-2 genome was performed using the COVID-19 ARTIC v3 Illumina library construction and sequencing protocol V.4 (10.17504/protocols.io.bgxjjxkn). The library was prepared and loaded on a R9.4.1/FLO-MIN106 flow cell, and sequenced with the MinION Mk1B (Oxford Nanopore Technology, ONT). Base-calling of the resulting FAST5 files was performed using Guppy (version 3.4.5)^12^, and the RAMPART software (v1.0.5) from the ARTIC Network^13^ was used to monitor sequencing in real-time. The ARTIC Network bioinformatics protocol was used for all genome assembly and variant calling steps^14^. Figure 4 illustrates the resultant SARS-CoV-2 genome organization and identified mutations. Two isolates of UAMS SARS-CoV-2 genome were identified and grouped into a clade^15^. Black circles and black squares in Fig. 4 identify the locations used for clade identification characterized by Global Initiative on Sharing All Influenza Data (GISAID)^15^. TrackViewer software was used for visualization^16^.

## Data Records

### COVID-19-AR image collection

Image data were extracted from the clinical PACS (Sectra AB, Linkoping, Sweden) at the University of Arkansas for Medical Sciences using Digital Imaging and Communications in Medicine (DICOM) query/retrieve software. All image data were de-identified and stored in DICOM standard format^17^ on TCIA as collection COVID-19-AR^18^.

### Clinical data structure

All clinical data were obtained from the Arkansas Clinical Data Repository (AR-CDR)^19^. The AR-CDR is a research data warehouse that provides a single and secure source of data for use in clinical and translational research; housing data for this project that are extracted from the EPIC (Epic Systems Inc, Verona, WI) electronic health record (EHR) system and legacy systems.

Data are provided on TCIA^18^ in a spreadsheet format (Microsoft excel) with one tab for patient data and a second for imaging study related data. Patient demographics, outcomes, co-morbidities and other clinical information are provided. Online-only Table 2 defines categorical variables (column headings) that are included.

### Genomic data structure

Viral genomes are stored in the GenBank FASTA format^20^ which stores both a nucleotide sequence and its corresponding quality scores. All required metadata, including collection dates, location, nucleotide sequence, amino acid sequences, and gene annotations, as described in the GenBank data model^21^ were provided for each sequence.

Data are available from the NCBI Sequence Read Archive^22^ and via direct links to each SARS-CoV-2 viral genome^23,24^. An NCBI BioProject was created (https://identifiers.org/bioproject:PRJNA643530) and also provides links to the viral genomes. Links to these data are also provided on TCIA^18^.

## Technical Validation

### Imaging and clinical data

All image data were processed using standard TCIA curation workflows. TCIA uses a standards-based approach for de-identification of images stored in the Digital Imaging and Communications in Medicine (DICOM) format. DICOM Standard PS3.15 2016a - Security and System Management Profiles^25^ defines how to correctly de-identify DICOM objects. The image data described in this data descriptor were de-identified using the “Basic Application Confidentiality Profile,” amended by inclusion of profile options: Clean Pixel Data Option (removal of information burned into the pixel data), Clean Descriptors Option (removal of PHI from data elements of type text or string), Retain Longitudinal With Modified Dates Option (all dates are shifted in a consistent manner by a random number so that relative temporal information is retained but absolute dates are masked), Retain Patient Characteristics Option (descriptive patient information is retained), Retain Device Identity Option (acquisition system information of potential scientific value is retained), and Retain Safe Private Option (retain scientifically valuable information stored in vendor private data elements) as is standard TCIA practice. Additional details on the de-identification process including the process for modifying dates by shifting using a random number are provided on the TCIA web site (wiki.cancerimagingarchive.net/display/Public/Submission+and+De-identification+Overview).

The TCIA curation team verifies completeness of the received collection, full removal of all PHI, proper labeling of all information to facilitate retrieval, and proper linkages among components of the collection^26–28^. TCIA curation procedures assure image quality and data integrity. TCIA uses the Posda open-source framework to implement its curation process^29,30^. Posda-based TCIA curation workflows ensure the scientific utility of data and eliminate protected health information. After Posda processing is complete, a data curator visually inspects every DICOM image.

Patient demographics, outcomes, and other clinical information were also validated during the TCIA curation process. Prior to this review, two of the authors (AB, FP) manually reviewed all clinical data for consistency, accuracy, and completeness, while SD and FP reviewed all image data and radiology reports. Images were reviewed on the clinical PACS and using TCIA’s radiology image viewer^31^.

### SARS-CoV-2 genome sequencing

To validate the ARTIC protocol for both experimental and computational methods, we included the Washington strain SARS-CoV-2 genomics RNA (2019-nCoV/USA-WA1/2020, MN985325.1) acquired from the American Type Culture Collection (ATCC) as the positive control and nuclease-free water as the negative control. The positive control and negative control were sequenced simultaneously with the clinical sequences and analyzed using the ARTIC protocol to obtain SARS-CoV-2 genomes. The SARS-CoV-2 genome sequence from the positive control showed no differences relative to the original genome sequence, MN985325.1. For the negative control, no viral sequence was obtained.

## Online-only Tables

**Online-only Table 1:** CT Acquisition Parameters for Each Image Series.

PATIENT ID	SERIES INSTANCE UID	SERIES DESCRIPTION	VENDOR	MODEL	KVP	MAS	KERNEL
COVID-19-AR-16406488	1.3.6.1.4.1.14519.5.2.1.9999.103.1313063663834745754311569016446	LOCATOR	PHILIPS	BRILLIANCE 64	120	107	B
COVID-19-AR-16406488	1.3.6.1.4.1.14519.5.2.1.9999.103.1383529064930736086410381663444	MERGED	PHILIPS	BRILLIANCE 64	120	190	A
COVID-19-AR-16406488	1.3.6.1.4.1.14519.5.2.1.9999.103.2145599909481308527014062204452	MPR, CORONALS, CORONAL	PHILIPS	BRILLIANCE 64	120	292	A
COVID-19-AR-16406488	1.3.6.1.4.1.14519.5.2.1.9999.103.2179137684384294959699673428806	3MM	PHILIPS	BRILLIANCE 64	120	284	B
COVID-19-AR-16406488	1.3.6.1.4.1.14519.5.2.1.9999.103.3320809190096644283982296275260	THINS	PHILIPS	BRILLIANCE 64	120	290	A
COVID-19-AR-16406488	1.3.6.1.4.1.14519.5.2.1.9999.103.7910615066871902088015121404116	SCOUT	PHILIPS	BRILLIANCE 64	120	50	NULL
COVID-19-AR-16406488	1.3.6.1.4.1.14519.5.2.1.9999.103.9151599194368935938086499337906	MPR, CORONALS, CORONAL	PHILIPS	BRILLIANCE 64	120	66	A
COVID-19-AR-16406490	1.3.6.1.4.1.14519.5.2.1.9999.103.2177696049178668428881815020900	1MM	PHILIPS	BRILLIANCE 64	120	224	B
COVID-19-AR-16406490	1.3.6.1.4.1.14519.5.2.1.9999.103.3011260124523077216718700620033	3MM	PHILIPS	BRILLIANCE 64	120	227	B
COVID-19-AR-16406490	1.3.6.1.4.1.14519.5.2.1.9999.103.4463588693392912664937903248239	LOCATOR	PHILIPS	BRILLIANCE 64	120	107	B
COVID-19-AR-16406490	1.3.6.1.4.1.14519.5.2.1.9999.103.5534834028017045447244258772980	MPR, 2MM, CORONAL	PHILIPS	BRILLIANCE 64	120	224	B
COVID-19-AR-16406490	1.3.6.1.4.1.14519.5.2.1.9999.103.6248221552676040679332389483766	MPR, 2MM, SAGITTAL	PHILIPS	BRILLIANCE 64	120	224	B
COVID-19-AR-16406490	1.3.6.1.4.1.14519.5.2.1.9999.103.7002876652027584491837379425496	SCOUT	PHILIPS	BRILLIANCE 64	120	50	NULL
COVID-19-AR-16406498	1.3.6.1.4.1.14519.5.2.1.9999.103.1045268951221473848204516633737	3X3S	PHILIPS	BRILLIANCE 64	120	137	A
COVID-19-AR-16406498	1.3.6.1.4.1.14519.5.2.1.9999.103.2001955707152085993458234797077	SURVIEW	PHILIPS	BRILLIANCE 64	120	30	NULL
COVID-19-AR-16406498	1.3.6.1.4.1.14519.5.2.1.9999.103.3217666904940907909280970082871	THINS	PHILIPS	BRILLIANCE 64	120	119	A
COVID-19-AR-16406498	1.3.6.1.4.1.14519.5.2.1.9999.103.9857888997538055942277915336788	MPR, CORONALS, CORONAL	PHILIPS	BRILLIANCE 64	120	149	A
COVID-19-AR-16406502	1.3.6.1.4.1.14519.5.2.1.9999.103.1051783119318881724733038231848	NULL	PHILIPS	BRILLIANCE 64	120	30	NULL
COVID-19-AR-16406502	1.3.6.1.4.1.14519.5.2.1.9999.103.1997701302520502863624920432442	MPR, CORONALS, CORONAL	PHILIPS	BRILLIANCE 64	120	486	A
COVID-19-AR-16406502	1.3.6.1.4.1.14519.5.2.1.9999.103.2610350961696176323235789206308	THINS	PHILIPS	BRILLIANCE 64	120	360	A
COVID-19-AR-16406502	1.3.6.1.4.1.14519.5.2.1.9999.103.3247091443582319833651607421612	3X3S	PHILIPS	BRILLIANCE 64	120	465	A
COVID-19-AR-16406502	1.3.6.1.4.1.14519.5.2.1.9999.103.1394664645301387326519009777216	SCOUT	PHILIPS	BRILLIANCE 64	120	50	NULL
COVID-19-AR-16406502	1.3.6.1.4.1.14519.5.2.1.9999.103.1892998694410640266017914996862	3MM	PHILIPS	BRILLIANCE 64	120	327	B
COVID-19-AR-16406502	1.3.6.1.4.1.14519.5.2.1.9999.103.2601334907811783908201006290089	SAG	PHILIPS	BRILLIANCE 64	120	482	B
COVID-19-AR-16406502	1.3.6.1.4.1.14519.5.2.1.9999.103.2762245170216765525769543134538	COR	PHILIPS	BRILLIANCE 64	120	482	B
COVID-19-AR-16406502	1.3.6.1.4.1.14519.5.2.1.9999.103.3176765705496966195239558171101	1MM	PHILIPS	BRILLIANCE 64	120	288	B
COVID-19-AR-16406502	1.3.6.1.4.1.14519.5.2.1.9999.103.6956828531926520076613048792831	LOCATOR	PHILIPS	BRILLIANCE 64	120	179	B
COVID-19-AR-16406503	1.3.6.1.4.1.14519.5.2.1.9999.103.1891326720085238222508091238377	LOCATOR	PHILIPS	BRILLIANCE 64	120	107	B
COVID-19-AR-16406503	1.3.6.1.4.1.14519.5.2.1.9999.103.2211844365912791623560241844461	MPR, 2MM, CORONAL	PHILIPS	BRILLIANCE 64	120	496	B
COVID-19-AR-16406503	1.3.6.1.4.1.14519.5.2.1.9999.103.2983719706541109694408140413419	1MM	PHILIPS	BRILLIANCE 64	120	496	B
COVID-19-AR-16406503	1.3.6.1.4.1.14519.5.2.1.9999.103.5994665633676297202362651387707	SCOUT	PHILIPS	BRILLIANCE 64	120	50	NULL
COVID-19-AR-16406503	1.3.6.1.4.1.14519.5.2.1.9999.103.6641081245395640654389668684507	MPR, 2MM, CORONAL	PHILIPS	BRILLIANCE 64	120	496	B
COVID-19-AR-16406503	1.3.6.1.4.1.14519.5.2.1.9999.103.7367402644669254408230687515387	MPR, 2MM, SAGITTAL	PHILIPS	BRILLIANCE 64	120	496	B
COVID-19-AR-16406503	1.3.6.1.4.1.14519.5.2.1.9999.103.9249202706125845809348987384271	3MM	PHILIPS	BRILLIANCE 64	120	489	B
COVID-19-AR-16406508	1.3.6.1.4.1.14519.5.2.1.9999.103.2126808530583880849010944819064	THINS	PHILIPS	BRILLIANCE 64	140	390	A
COVID-19-AR-16406508	1.3.6.1.4.1.14519.5.2.1.9999.103.2410724600234458651868224014834	MPR, 3MM, SAGITTAL	PHILIPS	BRILLIANCE 64	140	363	A
COVID-19-AR-16406508	1.3.6.1.4.1.14519.5.2.1.9999.103.2968245889624222918313373631650	VEN 3/3	PHILIPS	BRILLIANCE 64	140	390	A
COVID-19-AR-16406508	1.3.6.1.4.1.14519.5.2.1.9999.103.3133407702323681993506300660368	SURVIEW	PHILIPS	BRILLIANCE 64	120	50	NULL
COVID-19-AR-16406508	1.3.6.1.4.1.14519.5.2.1.9999.103.9822241255989549084465340283436	MPR, 3MM, CORONAL	PHILIPS	BRILLIANCE 64	140	363	A
COVID-19-AR-16406513	1.3.6.1.4.1.14519.5.2.1.9999.103.1125556634627600476591254063596	MPR, 2MM, SAGITTAL	PHILIPS	BRILLIANCE 64	120	357	B
COVID-19-AR-16406513	1.3.6.1.4.1.14519.5.2.1.9999.103.1217409760838081713893393545698	3MM	PHILIPS	BRILLIANCE 64	120	347	B
COVID-19-AR-16406513	1.3.6.1.4.1.14519.5.2.1.9999.103.1436621134085818814577012444458	LOCATOR	PHILIPS	BRILLIANCE 64	120	107	B
COVID-19-AR-16406513	1.3.6.1.4.1.14519.5.2.1.9999.103.2027423967398873929401454341345	1MM	PHILIPS	BRILLIANCE 64	120	332	B
COVID-19-AR-16406513	1.3.6.1.4.1.14519.5.2.1.9999.103.5577105015155286142296554220309	SCOUT	PHILIPS	BRILLIANCE 64	120	50	NULL
COVID-19-AR-16406513	1.3.6.1.4.1.14519.5.2.1.9999.103.7395060610648542248993326522585	MPR, 2MM, CORONAL	PHILIPS	BRILLIANCE 64	120	357	B
COVID-19-AR-16407187	1.3.6.1.4.1.14519.5.2.1.9999.103.1249103157349783283756782145243	3MM	PHILIPS	BRILLIANCE 64	120	285	B
COVID-19-AR-16407187	1.3.6.1.4.1.14519.5.2.1.9999.103.1250097654857344015449525195815	SCOUT	PHILIPS	BRILLIANCE 64	120	50	NULL
COVID-19-AR-16407187	1.3.6.1.4.1.14519.5.2.1.9999.103.2644204237118861651930136844707	1MM	PHILIPS	BRILLIANCE 64	120	320	B
COVID-19-AR-16407187	1.3.6.1.4.1.14519.5.2.1.9999.103.3232439442170564874652627681894	MPR, 2MM, SAGITTAL	PHILIPS	BRILLIANCE 64	120	476	B
COVID-19-AR-16407187	1.3.6.1.4.1.14519.5.2.1.9999.103.5890661550751826352331906011640	MPR, 2MM, CORONAL	PHILIPS	BRILLIANCE 64	120	476	B
COVID-19-AR-16424071	1.3.6.1.4.1.14519.5.2.1.9999.103.1974580327187246687236950465908	CORONAL	TOSHIBA	AQUILION ONE	120	600	FC14
COVID-19-AR-16424071	1.3.6.1.4.1.14519.5.2.1.9999.103.2409149706128320185107643633250	NULL	TOSHIBA	AQUILION ONE	120	80	FC02
COVID-19-AR-16424071	1.3.6.1.4.1.14519.5.2.1.9999.103.2529449006689786062134624053596	NULL	TOSHIBA	AQUILION ONE	120	600	FC08
COVID-19-AR-16424071	1.3.6.1.4.1.14519.5.2.1.9999.103.2879587690093359092672637396540	SAG-MIP	TOSHIBA	AQUILION ONE	120	600	FC18
COVID-19-AR-16424071	1.3.6.1.4.1.14519.5.2.1.9999.103.2958836596564507576636044623577	COR-MIP	TOSHIBA	AQUILION ONE	120	600	FC18
COVID-19-AR-16424071	1.3.6.1.4.1.14519.5.2.1.9999.103.3665557652341105345917977862537	VOL.	TOSHIBA	AQUILION ONE	120	598	FC14
COVID-19-AR-16424071	1.3.6.1.4.1.14519.5.2.1.9999.103.8099298483363937117888691775172	NULL	TOSHIBA	AQUILION ONE	120	100	FL04
COVID-19-AR-16424076	1.3.6.1.4.1.14519.5.2.1.9999.103.1810398886634679170890664861527	SURVIEW	PHILIPS	BRILLIANCE 64	120	50	NULL
COVID-19-AR-16424076	1.3.6.1.4.1.14519.5.2.1.9999.103.3176289021424588550057287940814	MPR, SAG, SAGITTAL	PHILIPS	BRILLIANCE 64	120	181	A
COVID-19-AR-16424076	1.3.6.1.4.1.14519.5.2.1.9999.103.3200638077112701536258849576914	3MM ART	PHILIPS	BRILLIANCE 64	120	256	A
COVID-19-AR-16424076	1.3.6.1.4.1.14519.5.2.1.9999.103.3445553567187475536742363555695	LOCATOR	PHILIPS	BRILLIANCE 64	120	60	B
COVID-19-AR-16424076	1.3.6.1.4.1.14519.5.2.1.9999.103.4902632275489398235861966976262	MPR, COR, CORONAL	PHILIPS	BRILLIANCE 64	120	181	A
COVID-19-AR-16424081	1.3.6.1.4.1.14519.5.2.1.9999.103.1202866610909920924014614282020	NULL	TOSHIBA	AQUILION ONE	120	120	FC02
COVID-19-AR-16424081	1.3.6.1.4.1.14519.5.2.1.9999.103.1242357114515717354004279220822	CORONAL	TOSHIBA	AQUILION ONE	120	577	FC14
COVID-19-AR-16424081	1.3.6.1.4.1.14519.5.2.1.9999.103.1640415325467854718227903291760	SAG-MIP	TOSHIBA	AQUILION ONE	120	577	FC18
COVID-19-AR-16424081	1.3.6.1.4.1.14519.5.2.1.9999.103.2816889689583137574982800530220	NULL	TOSHIBA	AQUILION ONE	120	578	FC08
COVID-19-AR-16424081	1.3.6.1.4.1.14519.5.2.1.9999.103.2990612607173722020813991533712	VOL.	TOSHIBA	AQUILION ONE	120	600	FC14
COVID-19-AR-16424081	1.3.6.1.4.1.14519.5.2.1.9999.103.3070688268039044675043591326270	COR-MIP	TOSHIBA	AQUILION ONE	120	577	FC18
COVID-19-AR-16424081	1.3.6.1.4.1.14519.5.2.1.9999.103.3227718533236302176693760881666	NULL	TOSHIBA	AQUILION ONE	120	100	FL04
COVID-19-AR-16424106	1.3.6.1.4.1.14519.5.2.1.9999.103.1294632132204621689199275698823	LOCATOR	PHILIPS	BRILLIANCE 64	120	107	B
COVID-19-AR-16424106	1.3.6.1.4.1.14519.5.2.1.9999.103.1337968298205629613875883947838	MPR, 2MM, CORONAL	PHILIPS	BRILLIANCE 64	140	392	B
COVID-19-AR-16424106	1.3.6.1.4.1.14519.5.2.1.9999.103.1407025794001213141292634932369	SCOUT	PHILIPS	BRILLIANCE 64	120	50	NULL
COVID-19-AR-16424106	1.3.6.1.4.1.14519.5.2.1.9999.103.2923735667052596182744752363702	LOCATOR	PHILIPS	BRILLIANCE 64	120	357	B
COVID-19-AR-16424106	1.3.6.1.4.1.14519.5.2.1.9999.103.3044444040739789054048814119686	3MM	PHILIPS	BRILLIANCE 64	140	392	B
COVID-19-AR-16424106	1.3.6.1.4.1.14519.5.2.1.9999.103.3109954242791307686680033847985	1MM	PHILIPS	BRILLIANCE 64	140	392	B
COVID-19-AR-16424106	1.3.6.1.4.1.14519.5.2.1.9999.103.3176178182875206042295330509711	MPR, 2MM, SAGITTAL	PHILIPS	BRILLIANCE 64	140	392	B
COVID-19-AR-16424111	1.3.6.1.4.1.14519.5.2.1.9999.103.1945985571970332122354076379479	SCOUT	PHILIPS	BRILLIANCE 64	120	50	NULL
COVID-19-AR-16424111	1.3.6.1.4.1.14519.5.2.1.9999.103.2247493262127869519535136350026	THINS	PHILIPS	BRILLIANCE 64	120	233	A
COVID-19-AR-16424111	1.3.6.1.4.1.14519.5.2.1.9999.103.2549562329375741315213884171315	ART	PHILIPS	BRILLIANCE 64	120	264	A
COVID-19-AR-16424111	1.3.6.1.4.1.14519.5.2.1.9999.103.2658549969641693933249595684963	VENOUS	PHILIPS	BRILLIANCE 64	120	251	B
COVID-19-AR-16424111	1.3.6.1.4.1.14519.5.2.1.9999.103.7535167363478796349781661725574	MPR, ART THINS, CORONAL	PHILIPS	BRILLIANCE 64	120	232	A
COVID-19-AR-16424120	1.3.6.1.4.1.14519.5.2.1.9999.103.1642103496752928743039756891965	LOCATOR	PHILIPS	BRILLIANCE 64	120	107	B
COVID-19-AR-16424120	1.3.6.1.4.1.14519.5.2.1.9999.103.1904769635104498805238494024754	MPR, CORONALS, CORONAL	PHILIPS	BRILLIANCE 64	120	257	A
COVID-19-AR-16424120	1.3.6.1.4.1.14519.5.2.1.9999.103.2260445148333933366931147945925	3MM	PHILIPS	BRILLIANCE 64	120	157	B
COVID-19-AR-16424120	1.3.6.1.4.1.14519.5.2.1.9999.103.2293729936636053121820651036299	MERGED	PHILIPS	BRILLIANCE 64	120	196	A
COVID-19-AR-16424120	1.3.6.1.4.1.14519.5.2.1.9999.103.3277495278639050841187868349649	THINS	PHILIPS	BRILLIANCE 64	120	344	A
COVID-19-AR-16424120	1.3.6.1.4.1.14519.5.2.1.9999.103.6145320650116093156990078440903	SCOUT	PHILIPS	BRILLIANCE 64	120	50	NULL
COVID-19-AR-16424120	1.3.6.1.4.1.14519.5.2.1.9999.103.7338941504449206447844285069771	MPR, CORONALS, CORONAL	PHILIPS	BRILLIANCE 64	120	69	A
COVID-19-AR-16434363	1.3.6.1.4.1.14519.5.2.1.9999.103.1003632015463262160636584880469	MPR, 2MM, SAGITTAL	PHILIPS	BRILLIANCE 64	120	496	B
COVID-19-AR-16434363	1.3.6.1.4.1.14519.5.2.1.9999.103.1236101208319372218942852361857	LOCATOR	PHILIPS	BRILLIANCE 64	120	107	B
COVID-19-AR-16434363	1.3.6.1.4.1.14519.5.2.1.9999.103.2416729812313429666071894674230	SCOUT	PHILIPS	BRILLIANCE 64	120	50	NULL
COVID-19-AR-16434363	1.3.6.1.4.1.14519.5.2.1.9999.103.2436533854031271427033323598745	1MM	PHILIPS	BRILLIANCE 64	120	446	B
COVID-19-AR-16434363	1.3.6.1.4.1.14519.5.2.1.9999.103.2662364088204600381919162376574	3MM	PHILIPS	BRILLIANCE 64	120	350	B
COVID-19-AR-16434363	1.3.6.1.4.1.14519.5.2.1.9999.103.3373936107781432675115934840637	MPR, 2MM, CORONAL	PHILIPS	BRILLIANCE 64	120	496	B
COVID-19-AR-16434368	1.3.6.1.4.1.14519.5.2.1.9999.103.1646745667311369211668677260549	MPR, COR, CORONAL	PHILIPS	BRILLIANCE 64	120	126	A
COVID-19-AR-16434368	1.3.6.1.4.1.14519.5.2.1.9999.103.1708257991762311974584287650907	SURVIEW	PHILIPS	BRILLIANCE 64	120	50	NULL
COVID-19-AR-16434368	1.3.6.1.4.1.14519.5.2.1.9999.103.2671888573218744472548048005648	3MM ART	PHILIPS	BRILLIANCE 64	120	229	A
COVID-19-AR-16434368	1.3.6.1.4.1.14519.5.2.1.9999.103.3160875498747276765846404039582	MPR, SAG, SAGITTAL	PHILIPS	BRILLIANCE 64	120	126	A
COVID-19-AR-16434369	1.3.6.1.4.1.14519.5.2.1.9999.103.1042469430372615640800562718184	MPR, COR, CORONAL	PHILIPS	BRILLIANCE 64	140	358	A
COVID-19-AR-16434369	1.3.6.1.4.1.14519.5.2.1.9999.103.1261448369979508555828528097882	3MM ART	PHILIPS	BRILLIANCE 64	140	341	A
COVID-19-AR-16434369	1.3.6.1.4.1.14519.5.2.1.9999.103.1332561364171962035371100950268	MPR, SAG, SAGITTAL	PHILIPS	BRILLIANCE 64	140	358	A
COVID-19-AR-16434369	1.3.6.1.4.1.14519.5.2.1.9999.103.5865625767365314589473897927812	SURVIEW	PHILIPS	BRILLIANCE 64	120	150	NULL
COVID-19-AR-16434369	1.3.6.1.4.1.14519.5.2.1.9999.103.8431873670990360959892036693263	LOCATOR	PHILIPS	BRILLIANCE 64	120	200	B
COVID-19-AR-16434411	1.3.6.1.4.1.14519.5.2.1.9999.103.1228878605404887156257817839736	3MM	PHILIPS	BRILLIANCE 64	120	216	B
COVID-19-AR-16434411	1.3.6.1.4.1.14519.5.2.1.9999.103.1789955008618778898747401848167	MPR, 2MM, CORONAL	PHILIPS	BRILLIANCE 64	120	249	B
COVID-19-AR-16434411	1.3.6.1.4.1.14519.5.2.1.9999.103.2288175523792386032423920366288	1MM	PHILIPS	BRILLIANCE 64	120	216	B
COVID-19-AR-16434411	1.3.6.1.4.1.14519.5.2.1.9999.103.2493472153521309498353481114386	THORAX	PHILIPS	BRILLIANCE 64	120	50	NULL
COVID-19-AR-16434411	1.3.6.1.4.1.14519.5.2.1.9999.103.2802505308029346417182455899783	LOCATOR	PHILIPS	BRILLIANCE 64	120	179	B
COVID-19-AR-16434411	1.3.6.1.4.1.14519.5.2.1.9999.103.9856987858107089484712500517091	MPR, 2MM, SAGITTAL	PHILIPS	BRILLIANCE 64	120	249	B
COVID-19-AR-16434453	1.3.6.1.4.1.14519.5.2.1.9999.103.1041339557012460371213363644773	MPR, 2MM, SAGITTAL	PHILIPS	BRILLIANCE 64	120	496	B
COVID-19-AR-16434453	1.3.6.1.4.1.14519.5.2.1.9999.103.2076457438901734987560753967101	3MM	PHILIPS	BRILLIANCE 64	120	496	B
COVID-19-AR-16434453	1.3.6.1.4.1.14519.5.2.1.9999.103.2192022583760413977255991809668	SCOUT	PHILIPS	BRILLIANCE 64	120	50	NULL
COVID-19-AR-16434453	1.3.6.1.4.1.14519.5.2.1.9999.103.2567763346491534859053724324331	MPR, 2MM, CORONAL	PHILIPS	BRILLIANCE 64	120	496	B
COVID-19-AR-16434453	1.3.6.1.4.1.14519.5.2.1.9999.103.3418955051608630050935500029279	LOCATOR	PHILIPS	BRILLIANCE 64	120	107	B
COVID-19-AR-16434453	1.3.6.1.4.1.14519.5.2.1.9999.103.7011144394500969894865045151607	1MM	PHILIPS	BRILLIANCE 64	120	496	B
COVID-19-AR-16445122	1.3.6.1.4.1.14519.5.2.1.9999.103.1956034301558123447424980594019	MPR, 2MM, CORONAL	PHILIPS	BRILLIANCE 64	120	343	B
COVID-19-AR-16445122	1.3.6.1.4.1.14519.5.2.1.9999.103.2156893184788557360959718358671	3MM	PHILIPS	BRILLIANCE 64	120	316	B
COVID-19-AR-16445122	1.3.6.1.4.1.14519.5.2.1.9999.103.3013881050239635135171412359738	LOCATOR	PHILIPS	BRILLIANCE 64	120	107	B
COVID-19-AR-16445122	1.3.6.1.4.1.14519.5.2.1.9999.103.3253966507871623107854383560515	MPR, 2MM, SAGITTAL	PHILIPS	BRILLIANCE 64	120	343	B
COVID-19-AR-16445122	1.3.6.1.4.1.14519.5.2.1.9999.103.3308141448836028549771371281500	1MM	PHILIPS	BRILLIANCE 64	120	442	B
COVID-19-AR-16445122	1.3.6.1.4.1.14519.5.2.1.9999.103.5906052974851959822811813399415	SCOUT	PHILIPS	BRILLIANCE 64	120	50	NULL
COVID-19-AR-16445138	1.3.6.1.4.1.14519.5.2.1.9999.103.1026569828019455865108676412494	1MM	PHILIPS	BRILLIANCE 64	120	496	B
COVID-19-AR-16445138	1.3.6.1.4.1.14519.5.2.1.9999.103.1059883416211444483187361429672	MPR, 2MM, SAGITTAL	PHILIPS	BRILLIANCE 64	120	496	B
COVID-19-AR-16445138	1.3.6.1.4.1.14519.5.2.1.9999.103.1840082189735209727773309569119	LOCATOR	PHILIPS	BRILLIANCE 64	120	357	B
COVID-19-AR-16445138	1.3.6.1.4.1.14519.5.2.1.9999.103.2188270175266453742370949922822	SCOUT	PHILIPS	BRILLIANCE 64	120	50	NULL
COVID-19-AR-16445138	1.3.6.1.4.1.14519.5.2.1.9999.103.2342323576795414470404804979111	MPR, 2MM, CORONAL	PHILIPS	BRILLIANCE 64	120	496	B
COVID-19-AR-16445138	1.3.6.1.4.1.14519.5.2.1.9999.103.2487441933538301467198700784216	LOCATOR	PHILIPS	BRILLIANCE 64	120	357	B
COVID-19-AR-16445138	1.3.6.1.4.1.14519.5.2.1.9999.103.3125812321537614785025843239681	3MM	PHILIPS	BRILLIANCE 64	120	496	B
COVID-19-AR-16445144	1.3.6.1.4.1.14519.5.2.1.9999.103.1101918195702862019498832306863	SCOUT	PHILIPS	BRILLIANCE 64	120	50	NULL
COVID-19-AR-16445144	1.3.6.1.4.1.14519.5.2.1.9999.103.1120265812971473099017869831733	3MM	PHILIPS	BRILLIANCE 64	120	332	B
COVID-19-AR-16445144	1.3.6.1.4.1.14519.5.2.1.9999.103.2449823669918640918906330283891	1MM	PHILIPS	BRILLIANCE 64	120	244	B
COVID-19-AR-16445144	1.3.6.1.4.1.14519.5.2.1.9999.103.2717796085351575017027848526455	MPR, 2MM, CORONAL	PHILIPS	BRILLIANCE 64	120	336	B
COVID-19-AR-16445144	1.3.6.1.4.1.14519.5.2.1.9999.103.3061713403254140279209387951208	LOCATOR	PHILIPS	BRILLIANCE 64	120	179	B
COVID-19-AR-16445144	1.3.6.1.4.1.14519.5.2.1.9999.103.4376895486643726196522872512352	MPR, 2MM, SAGITTAL	PHILIPS	BRILLIANCE 64	120	336	B
COVID-19-AR-16445168	1.3.6.1.4.1.14519.5.2.1.9999.103.1295108284294013545862428607034	NULL	TOSHIBA	AQUILION ONE	120	30	FL04
COVID-19-AR-16445168	1.3.6.1.4.1.14519.5.2.1.9999.103.1367270337350957115277262428872	VOL.	TOSHIBA	AQUILION ONE	120	507	FC14
COVID-19-AR-16445168	1.3.6.1.4.1.14519.5.2.1.9999.103.1925319293171912831908052353997	SAG-MIP	TOSHIBA	AQUILION ONE	120	507	FC18
COVID-19-AR-16445168	1.3.6.1.4.1.14519.5.2.1.9999.103.2512629351435775951698018710952	NULL	TOSHIBA	AQUILION ONE	120	311	FC08
COVID-19-AR-16445168	1.3.6.1.4.1.14519.5.2.1.9999.103.2703789419108154556272429780681	NULL	TOSHIBA	AQUILION ONE	120	100	FL04
COVID-19-AR-16445168	1.3.6.1.4.1.14519.5.2.1.9999.103.2874156157304654578612196414126	CORONAL	TOSHIBA	AQUILION ONE	120	507	FC14
COVID-19-AR-16445168	1.3.6.1.4.1.14519.5.2.1.9999.103.3024312905158332857389389755149	COR-MIP	TOSHIBA	AQUILION ONE	120	507	FC18
COVID-19-AR-16445168	1.3.6.1.4.1.14519.5.2.1.9999.103.3143985967861857451482349414304	NULL	TOSHIBA	AQUILION ONE	120	80	FC02
COVID-19-AR-16406488	1.3.6.1.4.1.14519.5.2.1.9999.103.2141196119890043114577038824717	MPR, MIP CORONAL, CORONAL	PHILIPS	BRILLIANCE 64	120	292	A
COVID-19-AR-16406488	1.3.6.1.4.1.14519.5.2.1.9999.103.2144551460887296005007561409842	MPR, MIP SAGITTAL, SAGITTAL	PHILIPS	BRILLIANCE 64	120	292	A
COVID-19-AR-16406488	1.3.6.1.4.1.14519.5.2.1.9999.103.2505549347573805412498876718016	MPR, SAGITTALS, SAGITTAL	PHILIPS	BRILLIANCE 64	120	292	A
COVID-19-AR-16406488	1.3.6.1.4.1.14519.5.2.1.9999.103.3985904991449630096370495463033	MPR, SAGITTALS, SAGITTAL	PHILIPS	BRILLIANCE 64	120	66	A
COVID-19-AR-16406498	1.3.6.1.4.1.14519.5.2.1.9999.103.3116385987553578422849617947833	MPR, SAGITTALS, SAGITTAL	PHILIPS	BRILLIANCE 64	120	149	A
COVID-19-AR-16406502	1.3.6.1.4.1.14519.5.2.1.9999.103.1243802757696273168583206288897	MPR, SAGITTALS, SAGITTAL	PHILIPS	BRILLIANCE 64	120	486	A
COVID-19-AR-16424111	1.3.6.1.4.1.14519.5.2.1.9999.103.1112979941533895161378164915661	MPR, ART THINS, SAGITTAL	PHILIPS	BRILLIANCE 64	120	232	A
COVID-19-AR-16424120	1.3.6.1.4.1.14519.5.2.1.9999.103.1520078341906815419782070061911	MPR, SAGITTALS, SAGITTAL	PHILIPS	BRILLIANCE 64	120	69	A
COVID-19-AR-16424120	1.3.6.1.4.1.14519.5.2.1.9999.103.1617153998178447255117636900766	MPR, MIP SAGITTAL, SAGITTAL	PHILIPS	BRILLIANCE 64	120	257	A
COVID-19-AR-16424120	1.3.6.1.4.1.14519.5.2.1.9999.103.2125975138199130863196410560131	MPR, SAGITTALS, SAGITTAL	PHILIPS	BRILLIANCE 64	120	257	A
COVID-19-AR-16424120	1.3.6.1.4.1.14519.5.2.1.9999.103.2654620323794848721017484705715	MPR, MIP CORONAL, CORONAL	PHILIPS	BRILLIANCE 64	120	257	A
COVID-19-AR-16434369	1.3.6.1.4.1.14519.5.2.1.9999.103.1130548927243681392488708223797	MPR, 3MM VENOUS, SAGITTAL	PHILIPS	BRILLIANCE 64	140	357	A
COVID-19-AR-16434369	1.3.6.1.4.1.14519.5.2.1.9999.103.2519936438025660304796393790285	MPR, 3MM VENOUS, CORONAL	PHILIPS	BRILLIANCE 64	140	357	A
COVID-19-AR-16406490	1.3.6.1.4.1.14519.5.2.1.9999.103.6454041364163432828942774299391	MPR, MIP CORONAL, CORONAL	PHILIPS	BRILLIANCE 64	120	224	B
COVID-19-AR-16406490	1.3.6.1.4.1.14519.5.2.1.9999.103.8462152423938566397512746335486	MPR, MIP SAGITTAL, SAGITTAL	PHILIPS	BRILLIANCE 64	120	224	B
COVID-19-AR-16406503	1.3.6.1.4.1.14519.5.2.1.9999.103.1628309102646834134392717256702	MPR, MIP CORONAL, CORONAL	PHILIPS	BRILLIANCE 64	120	496	B
COVID-19-AR-16406503	1.3.6.1.4.1.14519.5.2.1.9999.103.2937053094047337481065731246004	MPR, MIP SAGITTAL, SAGITTAL	PHILIPS	BRILLIANCE 64	120	496	B
COVID-19-AR-16406513	1.3.6.1.4.1.14519.5.2.1.9999.103.1221912485476522361486751061624	MPR, MIP SAGITTAL, SAGITTAL	PHILIPS	BRILLIANCE 64	120	357	B
COVID-19-AR-16406513	1.3.6.1.4.1.14519.5.2.1.9999.103.1250288205217959347315736859752	MPR, MIP CORONAL, CORONAL	PHILIPS	BRILLIANCE 64	120	357	B
COVID-19-AR-16407187	1.3.6.1.4.1.14519.5.2.1.9999.103.1528982991821002464422705189318	MPR, MIP SAGITTAL, SAGITTAL	PHILIPS	BRILLIANCE 64	120	476	B
COVID-19-AR-16407187	1.3.6.1.4.1.14519.5.2.1.9999.103.1732943752149219073964319031699	MPR, MIP CORONAL, CORONAL	PHILIPS	BRILLIANCE 64	120	476	B
COVID-19-AR-16424106	1.3.6.1.4.1.14519.5.2.1.9999.103.1925750867207838075957730984133	MPR, MIP CORONAL, CORONAL	PHILIPS	BRILLIANCE 64	140	392	B
COVID-19-AR-16424106	1.3.6.1.4.1.14519.5.2.1.9999.103.7696340962578260585340029691005	MPR, MIP SAGITTAL, SAGITTAL	PHILIPS	BRILLIANCE 64	140	392	B
COVID-19-AR-16434363	1.3.6.1.4.1.14519.5.2.1.9999.103.1538819633003614355819764041013	MPR, MIP CORONAL, CORONAL	PHILIPS	BRILLIANCE 64	120	496	B
COVID-19-AR-16434363	1.3.6.1.4.1.14519.5.2.1.9999.103.9160047948081592650199498659815	MPR, MIP SAGITTAL, SAGITTAL	PHILIPS	BRILLIANCE 64	120	496	B
COVID-19-AR-16434411	1.3.6.1.4.1.14519.5.2.1.9999.103.2368960827462708636022307923816	MPR, MIP CORONAL, CORONAL	PHILIPS	BRILLIANCE 64	120	249	B
COVID-19-AR-16434411	1.3.6.1.4.1.14519.5.2.1.9999.103.2827904341990418295571795907931	MPR, MIP SAGITTAL, SAGITTAL	PHILIPS	BRILLIANCE 64	120	249	B
COVID-19-AR-16434453	1.3.6.1.4.1.14519.5.2.1.9999.103.2551198062749871874092440921175	MPR, MIP SAGITTAL, SAGITTAL	PHILIPS	BRILLIANCE 64	120	496	B
COVID-19-AR-16434453	1.3.6.1.4.1.14519.5.2.1.9999.103.8738180234878749896828315102541	MPR, MIP CORONAL, CORONAL	PHILIPS	BRILLIANCE 64	120	496	B
COVID-19-AR-16445122	1.3.6.1.4.1.14519.5.2.1.9999.103.2026868491531231869896369397491	MPR, MIP SAGITTAL, SAGITTAL	PHILIPS	BRILLIANCE 64	120	343	B
COVID-19-AR-16445138	1.3.6.1.4.1.14519.5.2.1.9999.103.3170000692155667469747720952528	MPR, MIP SAGITTAL, SAGITTAL	PHILIPS	BRILLIANCE 64	120	496	B
COVID-19-AR-16445138	1.3.6.1.4.1.14519.5.2.1.9999.103.3396560157214296344263479145883	MPR, MIP CORONAL, CORONAL	PHILIPS	BRILLIANCE 64	120	496	B
COVID-19-AR-16445144	1.3.6.1.4.1.14519.5.2.1.9999.103.1324761838240810514791230979531	MPR, MIP SAGITTAL, SAGITTAL	PHILIPS	BRILLIANCE 64	120	336	B
COVID-19-AR-16445144	1.3.6.1.4.1.14519.5.2.1.9999.103.3015019491721075055583146735978	MPR, MIP CORONAL, CORONAL	PHILIPS	BRILLIANCE 64	120	336	B
COVID-19-AR-16424071	1.3.6.1.4.1.14519.5.2.1.9999.103.6890018054262867735676182523275	SAGITTAL	TOSHIBA	AQUILION ONE	120	600	FC14
COVID-19-AR-16424081	1.3.6.1.4.1.14519.5.2.1.9999.103.3082863524031259394121088988727	SAGITTAL	TOSHIBA	AQUILION ONE	120	577	FC14
COVID-19-AR-16445168	1.3.6.1.4.1.14519.5.2.1.9999.103.2878107231605395089975494769505	SAGITTAL	TOSHIBA	AQUILION ONE	120	507	FC14
COVID-19-AR-16406502	1.3.6.1.4.1.14519.5.2.1.9999.103.1487694806595663546521372428523	COR MIPS	PHILIPS	BRILLIANCE 64	120	482	B
COVID-19-AR-16406502	1.3.6.1.4.1.14519.5.2.1.9999.103.5151198345846291686710584892904	SAG MIPS	PHILIPS	BRILLIANCE 64	120	482	B
COVID-19-AR-16406508	1.3.6.1.4.1.14519.5.2.1.9999.103.1149707970768324988927353481463	BLAD DELAY	PHILIPS	BRILLIANCE 64	120	343	A
COVID-19-AR-16406508	1.3.6.1.4.1.14519.5.2.1.9999.103.2137754127603954659244348902818	KID DELAY	PHILIPS	BRILLIANCE 64	140	236	A
COVID-19-AR-16406508	1.3.6.1.4.1.14519.5.2.1.9999.103.2961003593311537513351737521162	KID DELAY	PHILIPS	BRILLIANCE 64	140	282	A
COVID-19-AR-16424076	1.3.6.1.4.1.14519.5.2.1.9999.103.2659455384040364495058728689944	ARTERIAL 1MM	PHILIPS	BRILLIANCE 64	120	143	A
COVID-19-AR-16424076	1.3.6.1.4.1.14519.5.2.1.9999.103.2827783204472120182975902604808	3MM VENOUS	PHILIPS	BRILLIANCE 64	120	303	A
COVID-19-AR-16424076	1.3.6.1.4.1.14519.5.2.1.9999.103.3848740547466334615853365390213	BLADDER DELAYS	PHILIPS	BRILLIANCE 64	120	127	A
COVID-19-AR-16424076	1.3.6.1.4.1.14519.5.2.1.9999.103.5268920000865709624934349889391	KIDNEY DELAYS	PHILIPS	BRILLIANCE 64	120	157	A
COVID-19-AR-16424111	1.3.6.1.4.1.14519.5.2.1.9999.103.1334670067423209424352089068742	ART THINS	PHILIPS	BRILLIANCE 64	120	224	A
COVID-19-AR-16424111	1.3.6.1.4.1.14519.5.2.1.9999.103.2071151560994292990303073020807	KIDNEY DELAYS	PHILIPS	BRILLIANCE 64	120	128	A
COVID-19-AR-16424111	1.3.6.1.4.1.14519.5.2.1.9999.103.2752888707095014258015882645716	VENOUS SAG	PHILIPS	BRILLIANCE 64	120	333	B
COVID-19-AR-16424111	1.3.6.1.4.1.14519.5.2.1.9999.103.2753522054513137489712053691987	VENOUS COR	PHILIPS	BRILLIANCE 64	120	333	B
COVID-19-AR-16434368	1.3.6.1.4.1.14519.5.2.1.9999.103.1763007215356339111810074482250	3MM VENOUS	PHILIPS	BRILLIANCE 64	120	218	A
COVID-19-AR-16434368	1.3.6.1.4.1.14519.5.2.1.9999.103.7535057571599271633703857282225	ARTERIAL 1MM	PHILIPS	BRILLIANCE 64	120	207	A
COVID-19-AR-16434368	1.3.6.1.4.1.14519.5.2.1.9999.103.8207427665530703343633156460160	KIDNEY DELAYS	PHILIPS	BRILLIANCE 64	120	119	A
COVID-19-AR-16434369	1.3.6.1.4.1.14519.5.2.1.9999.103.1010988897901027169638698048426	BLADDER DELAYS	PHILIPS	BRILLIANCE 64	120	498	A
COVID-19-AR-16434369	1.3.6.1.4.1.14519.5.2.1.9999.103.2571588625672387018784880321510	3MM VENOUS	PHILIPS	BRILLIANCE 64	140	357	A
COVID-19-AR-16434369	1.3.6.1.4.1.14519.5.2.1.9999.103.4973443140418665269179232742868	KIDNEY DELAYS	PHILIPS	BRILLIANCE 64	120	352	A
COVID-19-AR-16434369	1.3.6.1.4.1.14519.5.2.1.9999.103.5318942502561707464119028109836	ARTERIAL 1MM	PHILIPS	BRILLIANCE 64	140	358	A

**Online-only Table 2:** Clinical Data Categorical Variables.

Patient Data Tab	
Variable Name	Definition
PATIENT_ID	TCIA anonymized patient identifier that links imaging study variables to patient variables and imaging studies for the same patient
AGE	Patient age in years
SEX	Patient sex
RACE	Patient self-identified race category
ZIP	First 3 digits of the patient’s ZIP code for location identification
LATEST_BMI	Most recent body mass index measurement
LATEST WEIGHT	Most recent patient weight in pounds
LATEST HEIGHT	Most recent patient height in feet(‘) and inches(“)
COMORBIDITIES:	A binary vector of comorbidities defined as Y/N
tuberculosis	
systemic lupus erythmatosus	
rheumatoid arthritis	
extensive burns	
asplenia	
hyposplenia	
measles	
cytomegalovirus	
chicken pox	
herpes zoster	
malnutrition	
current pregnant	
chronic kidney disease	
diabetes type i	
diabetes type ii	
transplant	
hemodialysis pre diagnosis	
hemodialysis post diagnosis	
cancer	
COVID TEST POSITIVE	Confirmation of a positive COVID-19 PCR test result
TEST NAME	Specific test performed. This field may be NULL if the specific COVID-19 PCR test is unknown.
ICU Admit	Indication of ICU submission (Y/N)
# ICU admits	Number of times a patient was admitted to the ICU during the COVID-19 hospitalization
MORTALITY	Indication of patient death (Y/N)
**Imaging Studies Tab**	
PATIENT_ID	TCIA anonymized patient identifier that links imaging study variables to patient variables and imaging studies for the same patient
DIAG TO IMG STUDY (DAYS)	The number of days from the date of diagnosis to the date of the imaging study. All dates in the DICOM image objects have been shifted in time for patient privacy. The relative time between studies and the study order is preserved.
DIAGNOSIS TO IMAGING TIME (HRS)	The number of hours from the time of diagnosis to the time of the imaging study.
IMAGE STUDY DESCRIPTION	A brief description of the type of imaging study performed, e.g., XR CHEST AP PORTABLE (a portable chest radiograph)
STUDY MODALITY	DICOM defined Imaging modality, DX (digital radiograph), CR (computed radiography), CT (Computed Tomography)
FIO2 @ TIME OF IMG STUDY	Fraction of Inspired Oxygen at the time of the imaging study (in %). Range is 21% for room air to 100%. A NULL value is present if this measurement was not taken immediately prior to the imaging study.
RADIOLOGIST KEY FINDINGS	Key findings extracted from the signed reports created by board-certified radiologists
